# The Pattern of Retinal Ganglion Cell Loss in *OPA1*-Related Autosomal Dominant Optic Atrophy Inferred From Temporal, Spatial, and Chromatic Sensitivity Losses

**DOI:** 10.1167/iovs.16-20309

**Published:** 2017-01

**Authors:** Anna Majander, Catarina João, Andrew T. Rider, G. Bruce Henning, Marcela Votruba, Anthony T. Moore, Patrick Yu-Wai-Man, Andrew Stockman

**Affiliations:** 1 University College London, Institute of Ophthalmology, London, United Kingdom; 2 Moorfields Eye Hospital, London, United Kingdom; 3 Department of Ophthalmology, University of Helsinki, and Helsinki University Hospital, Helsinki, Finland; 4 School of Optometry and Vision Sciences, Cardiff University Cardiff, and Cardiff Eye Unit, University Hospital Wales, Cardiff, United Kingdom; 5 Ophthalmology Department, University of California-San Francisco School of Medicine, San Francisco, California, United States; 6 Wellcome Trust Centre for Mitochondrial Research, Institute of Genetic Medicine, Newcastle University and Newcastle Eye Centre, Royal Victoria Infirmary, Newcastle upon Tyne, United Kingdom

**Keywords:** dominant optic atrophy (DOA), *OPA1*, critical flicker fusion, magnocellular, koniocellular, parvocellular

## Abstract

**Purpose:**

Progressive retinal ganglion cell (RGC) loss is the pathological hallmark of autosomal dominant optic atrophy (DOA) caused by pathogenic *OPA1* mutations. The aim of this study was to conduct an in-depth psychophysical study of the visual losses in DOA and to infer any selective vulnerability of visual pathways subserved by different RGC subtypes.

**Methods:**

We recruited 25 patients carrying pathogenic *OPA1* mutations and age-matched healthy individuals. Spatial contrast sensitivity functions (SCSFs) and chromatic contrast sensitivity were quantified, the latter using the Cambridge Colour Test. In 11 patients, long (L) and short (S) wavelength–sensitive cone temporal acuities were measured as a function of target illuminance, and L-cone temporal contrast sensitivity (TCSF) as a function of temporal frequency.

**Results:**

Spatial contrast sensitivity functions were abnormal, with the loss of sensitivity increasing with spatial frequency. Further, the highest L-cone temporal acuity fell on average by 10 Hz and the TCSFs by 0.66 log_10_ unit. Chromatic thresholds along the protan, deutan, and tritan axes were 8, 9, and 14 times higher than normal, respectively, with losses increasing with age and S-cone temporal acuity showing the most significant age-related decline.

**Conclusions:**

Losses of midget parvocellular, parasol magnocellular, and bistratified koniocellular RGCs could account for the losses of high spatial frequency sensitivity and protan and deutan sensitivities, high temporal frequency sensitivity, and S-cone temporal and tritan sensitivities, respectively. The S-cone–related losses showed a significant deterioration with increasing patient age and could therefore prove useful biomarkers of disease progression in DOA.

Autosomal dominant optic atrophy (DOA; Online Mendelian Inheritance in Man [OMIM] 165500) is characterized by slowly deteriorating visual acuity, impaired color vision, and central field defects.^1–3^ The majority of patients with DOA carry a heterozygous mutation in the *OPA1* gene (OMIM 605290), which encodes for an inner mitochondrial membrane protein involved in myriad cellular functions, including mitochondrial biogenesis, the maintenance of the delicate balance between mitochondrial fusion and fission forces, and the regulation of apoptosis.^4–6^ Despite the ubiquitous cellular nature of mitochondria, the neuropathology is frequently limited to retinal ganglion cells (RGCs) within the inner retina, and the mechanisms that underlie this selective vulnerability have still not been fully defined.^1–3^ Affected individuals usually become symptomatic in childhood, and several optical coherence tomography studies have confirmed the early loss of RGCs.^7–11^ However, there is a marked inter- and intrafamilial variation in the severity and rate of progression of the disease, and the exact pattern and chronology of RGC loss are not fully understood.^2,10,11^

Visual information from the photoreceptors is transmitted by the bipolar cells to RGCs that in turn project their axons to the lateral geniculate nucleus and the superior colliculus. Three major visual pathways, namely, the parvo-, magno-, and koniocellular pathways, have been distinguished based on their RGC population and the nature of the transmitted visual information. The predominant RGC population is the midget cells that form the parvocellular pathway, which is thought to transmit achromatic visual information of high spatial, but low temporal resolution, and also red–green chromatic information produced by opponent long (L) and middle (M) wavelength–sensitive cone signals. Parasol cells constitute a smaller proportion of RGCs, and their L- and M-cone inputs form the magnocellular pathway, which transmits achromatic visual information of high temporal but low spatial resolution. Parallel processing in the retina and early visual pathway has been the subject of several excellent reviews.^12–19^ The relatively sparse small bistratified RGCs that form part of the koniocellular visual pathway are excited by short (S)-wavelength cones and inhibited by L- and M-cones, and they transmit a blue–yellow chromatic signal. The S-cones and their pathways have been the subject of several recent reviews.^20–23^

Unlike conventional clinical visual tests that distinguish relatively poorly between different visual pathways, advanced psychophysical methods can be designed that selectively test the performance of one individual pathway.^24^ However, it should be recognized that although concrete links are often made between these physiological pathways and psychophysical performance on various tasks, they are inevitably hypothetical and to some extent tenuous. In the context of DOA, psychophysical studies have focused mainly on color vision and have shown various deficits that tended to be most severe for discrimination along the tritan axis.^1,25–29^ There are two reports detailing deficits in the parvocellular and magnocellular pathways in DOA, with one showing abnormal achromatic contrast sensitivities and the other abnormal peripheral motion detection thresholds.^1,28^ Although both parvocellular and magnocellular pathways seem to be involved in visually affected patients, the severity and progression of deficits with increasing age remain unclear.

In this study, we used temporal, spatial, and chromatic psychophysical tests to assess the magno-, parvo-, and koniocellular pathways in patients with DOA caused by pathogenic *OPA1* mutations. The psychophysical temporal test protocols have previously been used in our laboratory to study various retinal pathologies.^30–33^ Our aim in this work was to characterize the visual losses, and to infer any selective losses in visual pathways subserved by different RGC types. The deficits observed for these functional visual parameters were also related to structural measures, namely, the thicknesses of the macular RGC and the peripapillary retinal nerve fiber layers (RNFL), and to the patient's age.

## Methods

### Participants

We included 25 patients with DOA from 15 independent families carrying 13 different pathogenic sequence variants in the *OPA1* gene. The genetic testing was performed in the Leeds Genetics Laboratory (St. James's University Hospital, Leeds, UK) and as part of two previously reported studies.^4,34^ All patients underwent achromatic spatial contrast sensitivity and color discrimination tests and the following ophthalmologic investigations: best-corrected visual acuity (BCVA) using the Early Treatment Diabetic Retinopathy Study (ETDRS) charts,^35^ static computer-automated white-on-white threshold perimetry (Swedish Interactive Testing Algorithm fast 30-2 Humphrey visual field [model 740; Humphrey Instruments, Dublin, CA, USA]), and optical coherence tomography (OCT) imaging of the macula and optic nerve head (see details below). The demographics, genotypes, and clinical characteristics of the DOA patients are given in Table 1. Eleven of the 25 DOA patients (P1–11) also performed an extended psychophysical test protocol that included L- and S-cone temporal vision tests. The genotypes and clinical data for all patients are given in Table 2. Normative spatial contrast sensitivity and chromatic sensitivity data were obtained from 15 individuals (aged 17–78 years old at the time of testing) with normal or corrected-to-normal visual acuities, and normal color vision as assessed by standard color vision tests (the Farnsworth-Munsell 100 hue test, The Hardy-Rand-Rittler [HRR] pseudoisochromatic test, and the Ishihara color vision test). These measurements were made as part of this study. Normative temporal acuity was also measured in 15 individuals (aged 17–67 years old at the time of testing). Three of them were studied as part of this study and 12 are taken from a previous study that used the same conditions as those used here.^31,33^ Similarly, normative temporal contrast sensitivity data (*n* = 8) were taken from a previous study.^31,33^

**Table 1:**
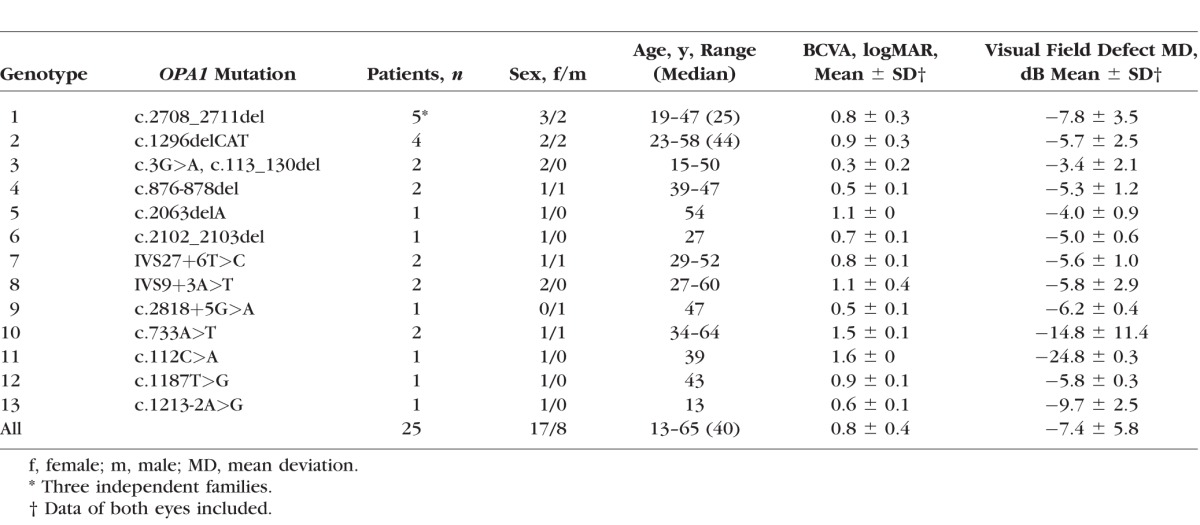
Demographics, Genotypes, and Clinical Data of the DOA Patients

**Table 2:**
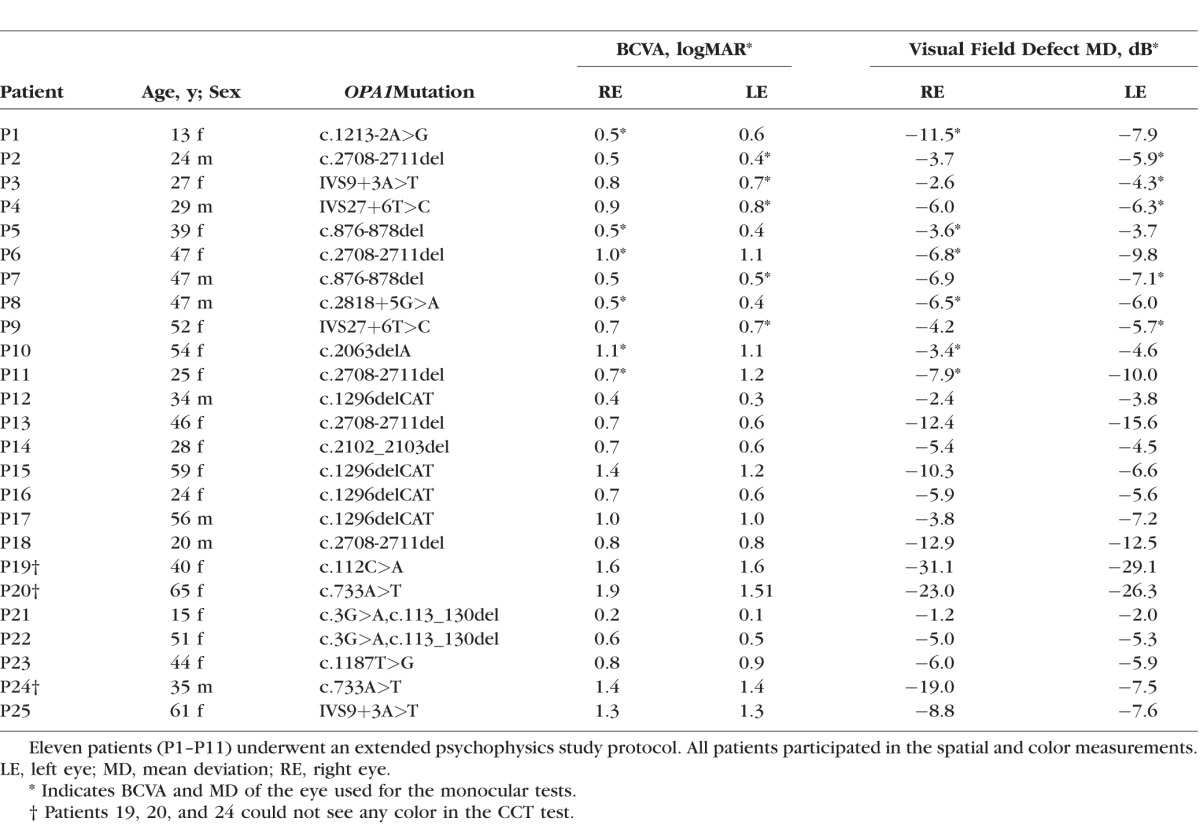
Characteristics of the DOA Patients

### L- and S-Cone Critical Flicker Fusion and L-Cone Modulation Sensitivity Measurements

L- and S-cone temporal acuities (critical flicker fusion, CFF) and L-cone temporal modulation sensitivity were measured using a Maxwellian-view optical system described in detail elsewhere.^33,36^ Two types of stimuli were used for the CFF measurements: The L-cone stimulus was a flickering, monochromatic, circular target with a diameter subtending 4° of visual angle and a wavelength of 650 nm. The target was presented in the center of a 9° diameter circular background field of 481 nm. The S-cone stimulus was also a flickering circular target of 4° diameter but of 440 nm in wavelength presented in the center of a 9° diameter background field of 620 nm in wavelength. For L-cone modulation sensitivity measurements, the time-averaged mean radiances of the 481-nm background and 650-nm target were fixed at 8.3 and 10.6 log quanta s^−1^deg^−2^, respectively. The observers varied the modulation of the sinusoidally flickering 650-nm target to find the modulation at each target frequency at which the flicker was just visible. In the CFF measurements, observers varied the frequency of the 650- or 440-nm target, which was sinusoidally flickering with a contrast of 92%, to find the frequency at which the flicker was just visible for a range of target radiances. Before each run, the observers light adapted to the background and target for 2 minutes. The observers viewed the stimuli monocularly with the eye they preferred (as indicated by the asterisk in Table 2) and interacted with the computer by means of an eight-button keypad as previously described.^33^ Each experiment was repeated two or three times on the same day. Details of these measurement techniques have been given elsewhere.^33^

### Achromatic Spatial Contrast Sensitivity Function (CSF)

The achromatic spatial contrast sensitivity function, the SCSF, was measured as a function of spatial frequency. The target stimuli were generated on a gamma-corrected Sony Trinitron monitor (model GDM F520; Sony Electronics, Inc., Park Ridge, NJ, USA) with 85-Hz frame rate and a resolution of 1600 × 1200 pixels connected to a DataPixx video processor (VPixx Technologies, Inc., Saint-Bruno, QC, Canada). The full screen subtended a visual angle of 39° × 29° at the test distance of 0.57 m. The experiments were performed at a constant mean luminance of 44.57 cd.m^−2^ as measured using a ColorCal calibration device (Cambridge Research Systems Ltd., Rochester, UK). The stimuli were circular centrally presented, horizontally oriented Gabor patterns with spatial standard deviations of 6° and spatial frequencies ranging from 0.25 to 16 cycles per degree (cyc/deg). The target duration was 500 ms, preceded and followed by 100-ms cosine-windowed onsets and offsets. The order of presentation was from low to high spatial frequencies. Thresholds were measured using a Yes/No staircase procedure. By means of a two-button keypad, observers indicated whether they could detect the Gabor pattern.

Stimulus contrast (Michelson contrast) was defined as (L_max_ − L_min_)/(L_max_ + L_min_), where L_max_ and L_min_ are the maximum and minimum luminances in the Gabor pattern, respectively. Contrast was adjusted following a one-up-one-down staircase procedure with a variable step size. For the first five reversals (changes in the direction of the staircase) the step size was 0.3 log units, after which the step size was reduced to 0.2 log units for two more reversals and then finally to 0.1 log unit for the last four reversals. A single run required a total of nine reversals, with the contrast “threshold” taken as the average of the last six reversals. Contrast sensitivity is the reciprocal of the contrast threshold. The spatial contrast sensitivity measurements lasted approximately 20 minutes for each observer. The stimuli were viewed binocularly with appropriate corrections if needed.

### Modeling Contrast Sensitivity Functions

In order to characterize the temporal and spatial contrast sensitivities, and how they differ between patients and control subjects, we fitted a cubic function in log–log coordinates, log_10_(*S*) = *α* log_10_(*f*) + *β* log_10_(*f*) + *γ* log_10_(*f*) + *δ*; where *S* is temporal or spatial contrast sensitivity, *f* is the frequency (Hz for temporal, and cyc/deg for spatial), and *α*, *β*, *γ*, and *δ* are constants. The cubic function here has no theoretical basis, but was chosen for its simplicity and because it provided a good fit to both the temporal and the spatial contrast threshold data for both the normal controls and the patients and therefore could be used to parameterize the contrast sensitivity functions.

### Chromatic Discrimination

Chromatic discrimination was tested using the Trivector test procedure implemented as part of the Cambridge Colour Test (CCT) (v1.5; Cambridge Research Systems Ltd.). The test was performed using a gamma-corrected Sony FD Trinitron color monitor (model GDM-F500R) connected to a VSG 2/5 visual stimulus generator (Cambridge Research Systems Ltd.) with 800- by 600-pixel resolution. The cathode ray tube (CRT) phosphors were measured, in CIE 1976 u′v′ chromaticity coordinates with a ColorCal photometer (Cambridge Research Systems Ltd.): red phosphor (R) u′ = 0.416; v′ = 0.522 (*x* = 0.610, *y* = 0.340); green phosphor (G) u′ = 0.117; v′ = 0.559 (*x* = 0.280, *y* = 0.595); blue phosphor (B) u′ = 0.159; v′ = 0.177 (*x* = 0.142, *y* = 0.070). The visual stimuli consisted of letter C targets presented on a background of neutral chromaticity (CIE 1976 coordinates u′ = 0.1977, v′ = 0.4689). The background and the target consisted of small circles of variable size and luminance (10 equal steps between 8.0 and 18.0 cd.m^−2^).

The test conditions were modified to be appropriate for observers with markedly reduced visual acuity. The viewing distance was set so that the gap in the letter C opening subtended 5° instead of 1° of visual angle. (For our system, this corresponded to a viewing distance of 62.6 cm.) Thresholds were measured along the three dichromat confusion lines, protan, deutan, and tritan, but the highest target saturation was increased to 1600 × 10^−4^ u′v′ units and the number of steps from 6 to 10 in order to maintain the standard unit difference between the steps. The time allowed for the subject to respond was increased from the standard 8 to 20 seconds. The test was then run using a standard one-up, one-down psychophysical staircase procedure with six reversals.^37^ The observers were instructed to respond to the gap position (four-alternative forced choice) by means of a four-button keypad. The test was performed binocularly with appropriate near corrections if needed.

The CCT depends on commercial software and hardware that uses a derivation of the 2° CIE standard observer data to generate the test stimuli and interpret the results. Although the 2° observer is plausible for normal control measurements, since normal observers used central vision and stimuli with gaps of 1°, it is not ideal for DOA patients who use larger targets and who can suffer from central scotomas. Unfortunately, the CCT cannot be modified for a larger field. Failures to isolate an aberrant cone response, however, should decrease thresholds, not increase them—as we find.

### Structural Studies

The Spectralis platform (Heidelberg Engineering Ltd., Heidelberg, Germany) was used for macular SD-OCT imaging. Automated segmentation and thickness analyses were performed for perifoveal volumetric retinal B-scans using the Heidelberg Engineering segmentation tool, included in the Spectralis Glaucoma Module software (version 6.0). The combined thickness of the ganglion cell layer (GCL) and inner plexiform layer (IPL) was recorded as mean of the four sectors of the inner ring (between 1 and 3 mm in diameter) of the nine macular ETDRS subfields as described elsewhere.^38^ Normative data for the GCL-IPL thickness was generated from SD-OCT images of 48 healthy eyes of 48 subjects.^38^ Peripapillary RNFL thickness measurements were performed using the Cirrus HD-OCT 4000 (Carl Zeiss Meditec, Inc., Dublin, CA, USA) platform. The mean RNFL thickness was recorded.

### Statistical Analyses

Mann-Whitney *U* independent samples test was used for comparison of distribution of continuous variables in DOA and normal observers, and in males and females, as indicated. Pearson product–moment correlation coefficient was used for the analysis of statistical dependence between various variables indicated in the Results section. Temporal functions were analyzed in DOA P1 to P11 within the data for the eyes used for the monocular temporal tests. All other variables were studied in the entire DOA study group. Binocular test data (CSF and CCT) were compared with the monocular data of the eye with the better BCVA. Monocular tests were analyzed for each eye separately.

The study was approved by local ethics committees at Moorfields Eye Hospital and at University College London and conformed to the standards set by the Declaration of Helsinki. All subjects or their parents signed informed consent forms.

## Results

### L-Cone Temporal Functions

**Figure 1:**
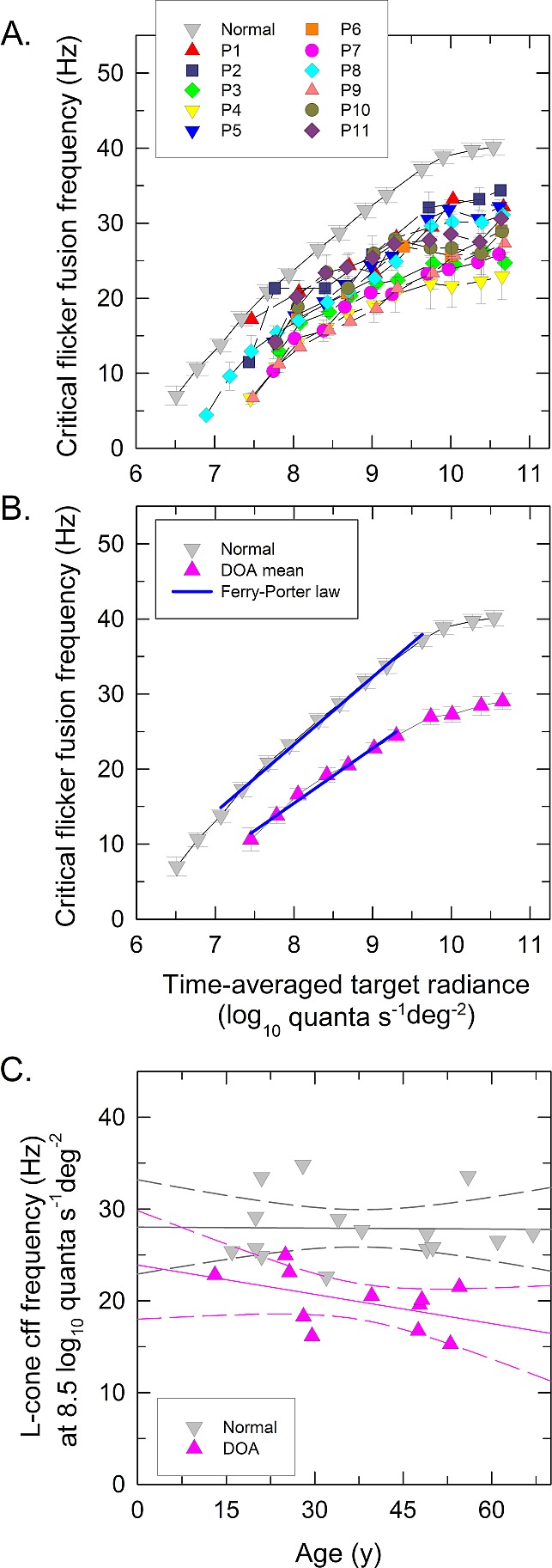
L-cone critical flicker fusion. (**A**, **B**) L-cone critical flicker frequencies (CFF) measured on a 481-nm background of 8.3 log_10_ quanta s^−1^deg^−2^ and plotted as a function of the mean log_10_ radiance of a 650-nm flickering target. (**A**) Data are plotted as mean ± 1 SEM for each DOA patient indicated with *individually colored symbols* and as *gray symbols* for the mean normal observer. (**B**) The *pink symbols* denote the mean ± 1 SEM across all DOA patients (except for the lowest two radiances that could be detected only by six and nine patients, respectively), and *gray symbols* again denote the mean of 15 normal observers (±1 SEM across observers). The Ferry-Porter slopes of mean CFF for DOA and normal observers are indicated by the *blue lines*. (**C**) The L-cone CFF frequencies at 8.5 log_10_ quanta s^−1^deg^−2^ plotted as a function of observer age for normal (*gray symbols*) and DOA patients (*pink symbols*). The *solid gray and pink lines* show the best fits for linear regression for normal and DOA patients, respectively—the *dashed lines*, the 95% confidence interval for the best-fitting linear regressions.

Figure 1A shows L-cone CFF (temporal acuity) data for the 11 DOA (P1–P11) and 15 normal observers plotted as a function of log_10_ target radiance. The mean data for 15 normal observers are shown by gray symbols, and the individual data for each DOA patient are shown by the colored symbols, as means ± 1 SEM. In the normal observers, the mean lowest target radiance at which flicker was seen was 6.6 log_10_ quanta s^−1^deg^−2^, after which the CFF rises until it approaches a plateau near 40 Hz. Most DOA patients are unable to see the flicker at such a low radiance and require, on average, a radiance of 7.4 log_10_ quanta s^−1^deg^−2^ before flicker can be observed. For them the asymptotic upper bound on flicker rate is highly variable, ranging from 24 to 35 Hz.

The mean data (±1 SEM across observers) for normal and DOA observers are shown in Figure 1B with gray and pink symbols, respectively. Over two and half log_10_ units the normal CFF increases as an approximately linear function of log_10_ radiance with a slope of 9.2 Hz per decade as shown by the blue line fitted to the mean normal data. This behavior is known as obedience to the Ferry-Porter law.^39,40^ For the DOA patients (pink triangles in Fig. 1B), the Ferry-Porter slope, also shown by a blue line, is 7.3 Hz per decade. Slope fits were also made to the individual DOA data over the range of CFFs consistent with the Ferry-Porter law and are tabulated in Table 3.

**Table 3:**
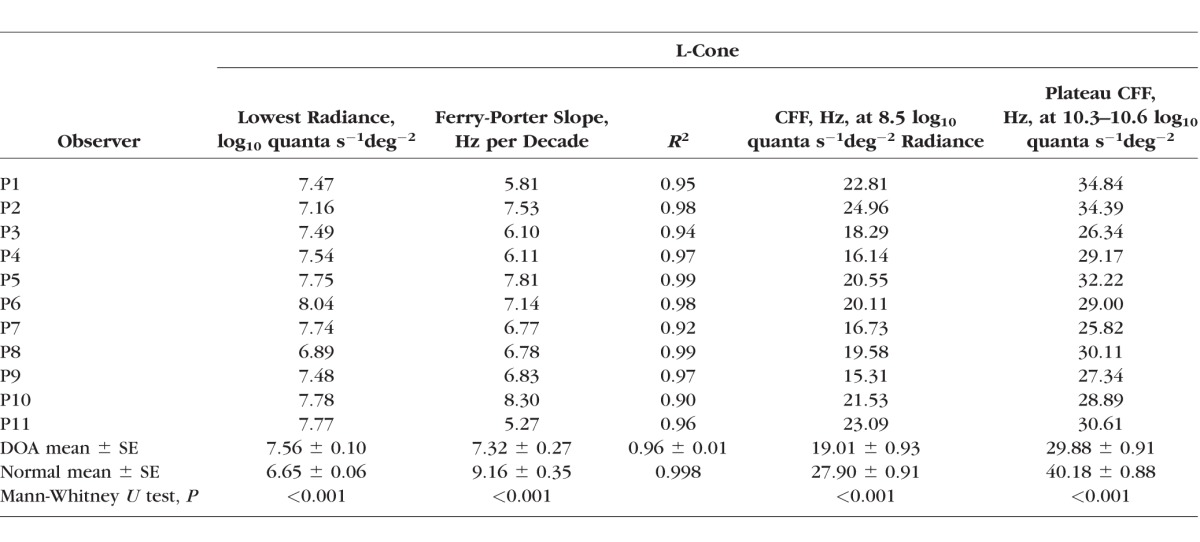
L-Cone Critical Flicker Fusion Variables

Figure 1C shows the L-cone CFF obtained at 8.5 log_10_ quanta s^−1^deg^−2^ plotted as a function of observer age for normal (gray symbols) and DOA observers (pink symbols). The solid gray and pink lines show the best fits for linear regression for normal and DOA observers, respectively, and the dashed lines indicate the ±95% confidence interval for the fits. Over the range of ages observed, L-cone CFF did not show any significant age-related variation either for the DOA or for the normal observers (Pearson's *r* = −0.474, *P* = 0.140 for DOA observers, and *r* = −0.018, *P* = 0.950 for normal observers). For the DOA observers (see Table 3), the L-cone plateau showed weakly significant age-related decreases (Pearson's *r* = −0.596, *P* = 0.053), and the Ferry-Porter slope weakly significant increases (*r* = 0.613, *P* = 0.045 for the Ferry-Porter slope).

Table 3 summarizes the CFF data and the Ferry-Porter fits: Column 2 shows the lowest detected target radiance and the mean value (±SEM) for 11 DOA patients, and the mean value (±SEM) for the normal observers. Columns 3 and 4 show the best-fitting Ferry-Porter slope together with the *R^2^* value for the fit; columns 5 and 6 show the CFF obtained at 8.5 log_10_ quanta s^−1^deg^−2^ interpolated from the Ferry-Porter slope together with the plateau frequency.^33^ Dominant optic atrophy patients first detected flicker at the mean radiance eight times (0.91 log unit) more intense than that for normal observers. In all DOA patients, CFF followed the Ferry-Porter law over a radiance range similar to that of normal observers, but in all patients the Ferry-Porter slope was shallower. This and the overall loss of sensitivity gives a mean reduction in CFF of 8.89 ± 0.93 Hz (mean ± SE) at 8.5 log_10_ quanta s^−1^deg^−2^. The plateau frequency was considerably lower in patients compared to normal observers (means of 29.88 and 40.18 Hz, respectively).

**Figure 2:**
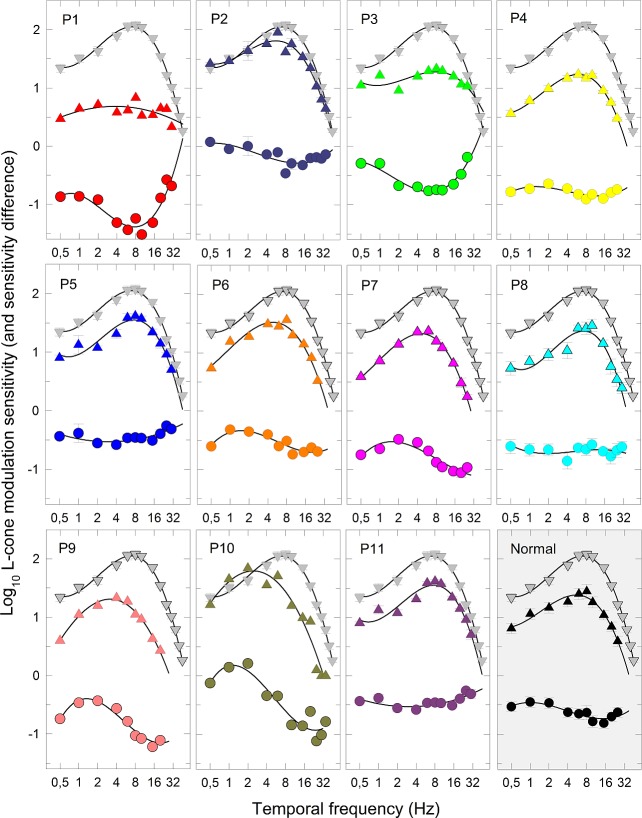
L-cone modulation sensitivity. Each image shows, for a single DOA patient, the log_10_ L-cone modulation sensitivities measured using a sinusoidally modulated, 650-nm 4° target with a mean radiance of 10.3 log_10_ quanta s^−1^deg^−2^ superimposed on a 9°, 481-nm background of 8.3 log_10_ quanta s^−1^deg^−2^ and plotted as a function temporal frequency colored triangles. (The *gray symbols* in each image show the mean normal data set for comparison.) The 12th (*gray*) image shows the mean DOA data (*black triangles*). The *error bars* are ±1 SEM either between runs for the individual patients, or between observers for the mean data. In the *lower part* of the first 11 images, the differences in log_10_ sensitivity between each DOA patient and the normal mean data (*n* = 8) are shown as *circles*. The 12th image shows the differences between the mean data (*filled circles*). The *black curves* are the fits of the cubic polynomial model described in Methods.

Figure 2 shows the logarithm of L-cone temporal modulation sensitivity as a function of temporal frequency measured with 4° targets at a time-averaged 650-nm target radiance of 10.6 log_10_ quanta s^−1^deg^−2^ for all observers. The mean data for 8 normal observers are shown in each image as gray triangles, while those for each of the 11 DOA patients are shown in the first 11 images as colored triangles. The mean data for the DOA patients are shown in the bottom right-hand image as filled triangles. The sensitivity differences between the DOA and mean normal data are shown in each image by the circles. The fits of the cubic polynomial model (described in Methods) are shown as black curves. The fitting parameters are given in Table 4.

**Table 4:**
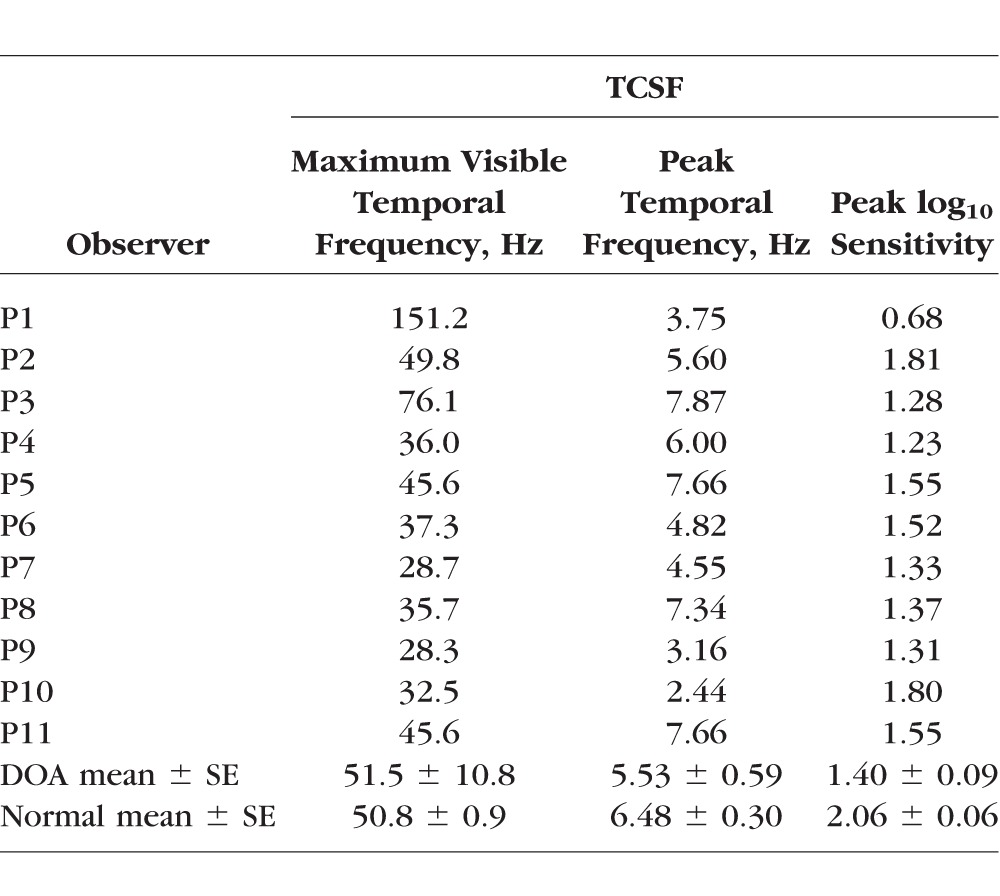
Peak and Maximal Sensitivity and Highest Visible TCSF Frequency

The mean L-cone modulation sensitivity function for normal observers peaks in sensitivity at 6.48 ± 0.30 Hz and falls off in sensitivity at both low and high temporal frequencies.^31–33^ This shape of sensitivity function is known as a band-pass function; the decline in sensitivity at low temporal frequencies is often attributed to surround antagonism.^41,42^ The temporal contrast sensitivity functions for all but one of the DOA patients (P1) were also band-pass in shape with a lower peak to the normal function (mean ± SEM: 5.53 ± 0.59 Hz), but they showed a mean peak sensitivity loss of 0.66 log_10_ units (mean ± SEM) (see image 12). The mean loss, shown in image 12, is roughly independent of temporal frequency. However, there are individual differences. The band-pass peaks for P9 and P10 are shifted to lower temporal frequencies and therefore showed bigger losses at high temporal frequencies. And the cone temporal contrast sensitivities (TCSFs) for P1 and P3 were fairly flat, so that the sensitivity relative to normal observers increased at higher temporal frequencies.

In conclusion, L-cone TCSFs were characterized by reduced temporal acuity or CFF, a mild but significant reduction in the Ferry-Porter CFF slope, and a general loss of sensitivity. L-cone temporal acuity showed weak age-related effects in terms of an increase in Ferry-Porter slope and a decrease in the plateau frequency, but CFF was approximately constant at middle intensities (see Fig. 1C).

### S-Cone Temporal Function—Critical Flicker Fusion

**Figure 3:**
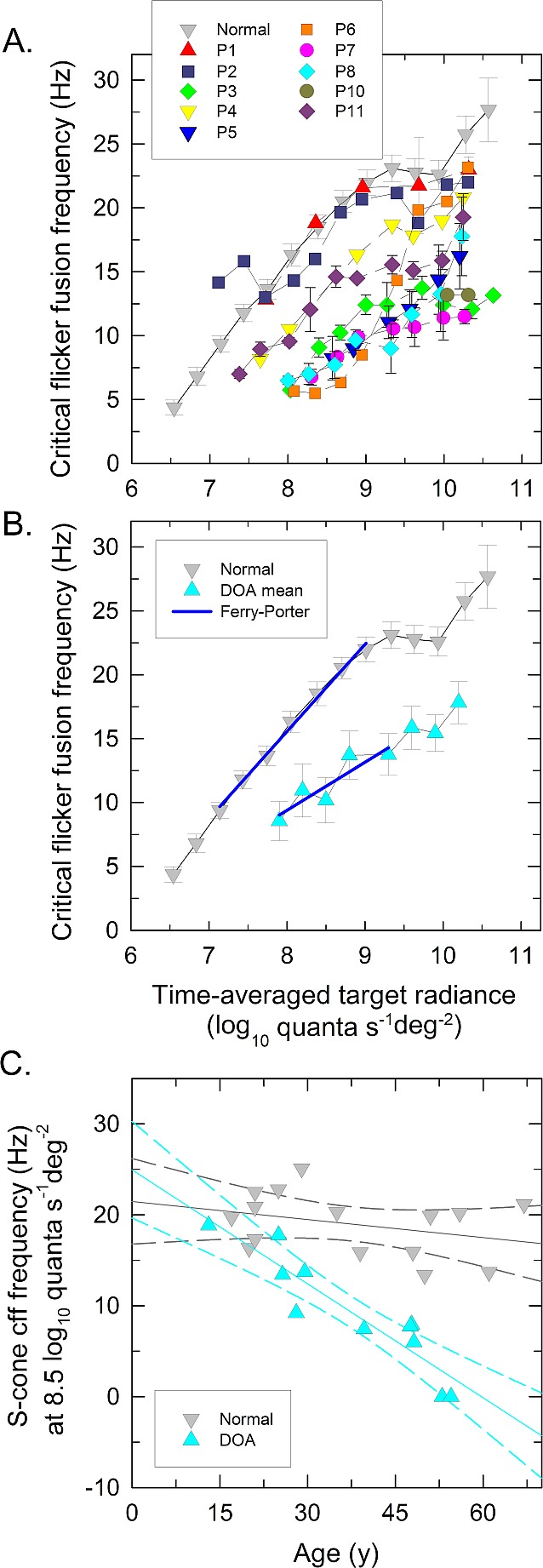
S-cone critical flicker fusion. (**A**, **B**) S-cone critical flicker frequencies (CFF) measured on a 9° 620-nm background of 11.41 log_10_ quanta s^−1^deg^−2^ plotted as a function of the mean log_10_ radiance of a 440-nm flickering target. (**A**) Data are plotted as mean ± 1 SEM for each DOA patient indicated with *individually colored symbols* and as *gray symbols* for the mean normal observer. (**B**) Mean DOA data with ±1 SEM across DOA patients indicated with *cyan symbols*. The *gray symbols* denote to the mean normal data again with ±1 SEM across 15 observers. The Ferry-Porter slopes of mean CFF for DOA and normal observers are indicated with *blue lines*. (**C**) The S-cone CFF frequencies at 8.5 log_10_ quanta s^−1^deg^−2^ plotted as a function of observer age, and the best fits for linear regression (*solid lines*) and 95% confidence intervals (*dashed lines*), for DOA patients (*cyan symbols* and *lines*) and for normal observers (*gray symbols* and *lines*).

Figures 3A and 3B show S-cone CFF data plotted as a function target radiance, and Table 5 tabulates CFF characteristics: the lowest detected target radiance, the Ferry-Porter slope, and the CFF frequency at 8.5 log_10_ quanta s^−1^deg^−2^ target radiance, for DOA and normal observers as in Table 3 but for S-cones. The data for 15 normal observers are again shown with gray symbols as mean ± 1 SEM. In the normal observers, S-cone CFF starts to rise above a radiance of 6.5 log_10_ quanta s^−1^deg^−2^ until reaching a plateau of approximately 22 Hz at 9.0 log_10_ quanta s^−1^deg^−2^. The linear relation between CFF and radiance, consistent with the Ferry-Porter law, is 7.3 Hz per log_10_ quanta s^−1^deg^−2^ for normal observers at radiances below the plateau. (The rise after 9.9 log_10_ quanta s^−1^deg^−2^ is due to M-cones becoming more sensitive than S-cones at high target radiances.^33,43^)

**Table 5:**
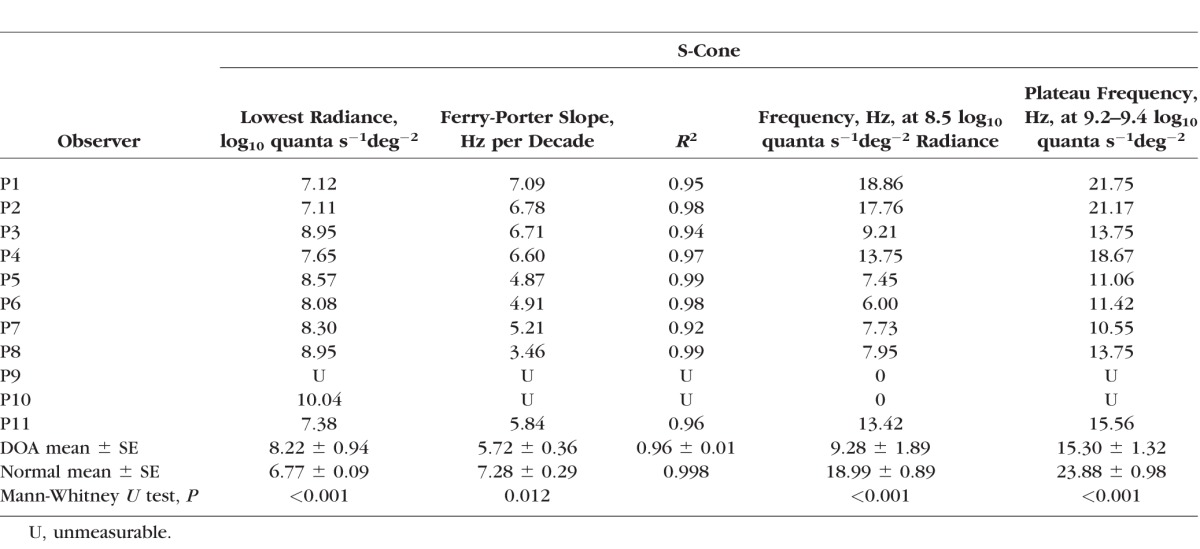
S-Cone Critical Flicker Fusion Variables

The data for each DOA patient are presented with individually colored symbols in Figure 3A. The data for S-cone CFF was severely abnormal for the oldest DOA patients (P9 and P10, 52 and 54 years old, respectively), so that P9 could not detect any flicker with a 440-nm target and P10 detected flicker only at the highest radiance (a radiance for which, in normal observers, M-cones are more sensitive than S-cones). The youngest DOA patients, in contrast, had normal Ferry-Porter slopes and near normal CFF frequencies at 8.5 log_10_ quanta s^−1^deg^−2^ as shown in Figure 3C where the S-cone CFF frequencies at 8.5 log_10_ quanta s^−1^deg^−2^ are plotted as a function of observer age for normal (gray symbols) and DOA (cyan symbols) observers. S-cone CFF at 8.5 log_10_ quanta s^−1^deg^−2^ showed a significant age-related variation for the DOA patients (Pearson's *r* = −0.920, *P* < 0.001) but not for the normal observers (Pearson's *r* = −0.326, *P* = 0.236). Also S-cone plateau frequency at 9.2 to 9.4 log_10_ quanta s^−1^deg^−2^ target radiance and the lowest detected radiance showed significant age correlation (Pearson's *r* = −0.838, *P* = 0.005 for plateau and Pearson's *r* = 0.667, *P* = 0.050 for lowest radiance). (Severely impaired S-cone temporal acuity associated with lowered S- and M-cone–related chromatic sensitivities as will be presented in detail later on). The mean CFF data for the DOA patients are shown in Figure 3B (cyan triangles).

### Achromatic Spatial Contrast Sensitivity Function

Figure 4 and Figure 5A show achromatic SCSFs, that is, sensitivity plotted in log_10_ units as a function of spatial frequency (cyc/deg) plotted on a logarithmic scale. The mean data for 15 normal observers are shown with gray symbols and have a peak mean contrast sensitivity of 2.19 ± 0.03 log_10_ (mean ± SEM) at a mean spatial frequency of 2.18 ± 0.15 cyc/deg (mean ± SEM). Like the TCSF, the SCSF also has a band-pass shape with losses at lower spatial frequencies probably resulting from surround inhibition.^41,44,45^ The response at the highest spatial frequencies is limited jointly by the modulation transfer function of the optics and the size of the receptive field center, so that only neurons with the smallest receptive fields are able to respond to the highest frequencies.^46–48^

**Figure 4:**
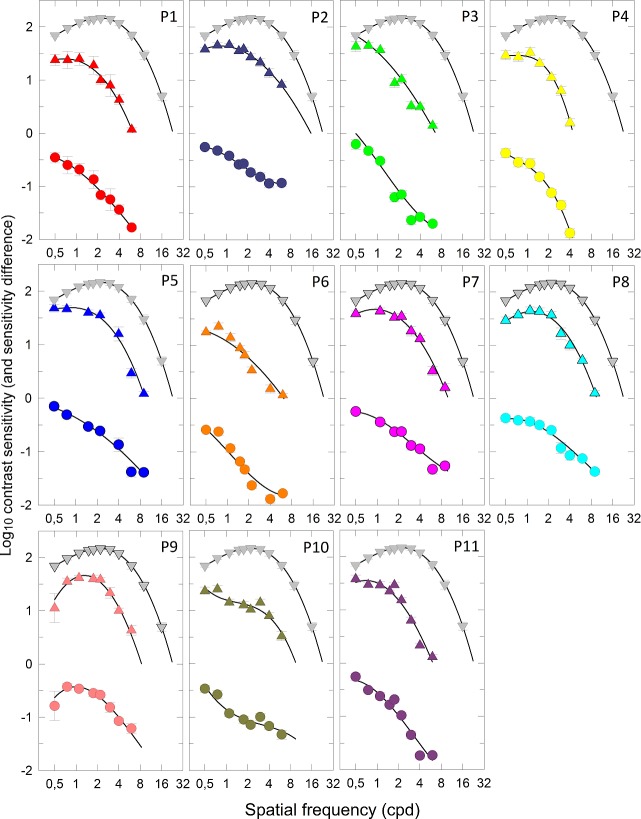
Achromatic spatial contrast sensitivity functions (SCSFs), expressed as log_10_ sensitivity as a function of spatial frequency (cycles per degree, cyc/deg—logarithmic axis), are indicated with *colored triangles* for (**A**) the 11 individual DOA patients who also participated in the temporal measurements and (**B**) the 14 individual DOA patients who did not, in separate images together with normal data (*inverted gray triangles*). The difference in sensitivity between each DOA patient and normal is also indicated in each image by *colored circles*. The *symbols* and *error bars* are mean ± 1 SEM across normal observers, or across repeated runs of individual DOA patients. The *black curves* are fits of a cubic polynomial model, as described in the text.

**Figure 4:**
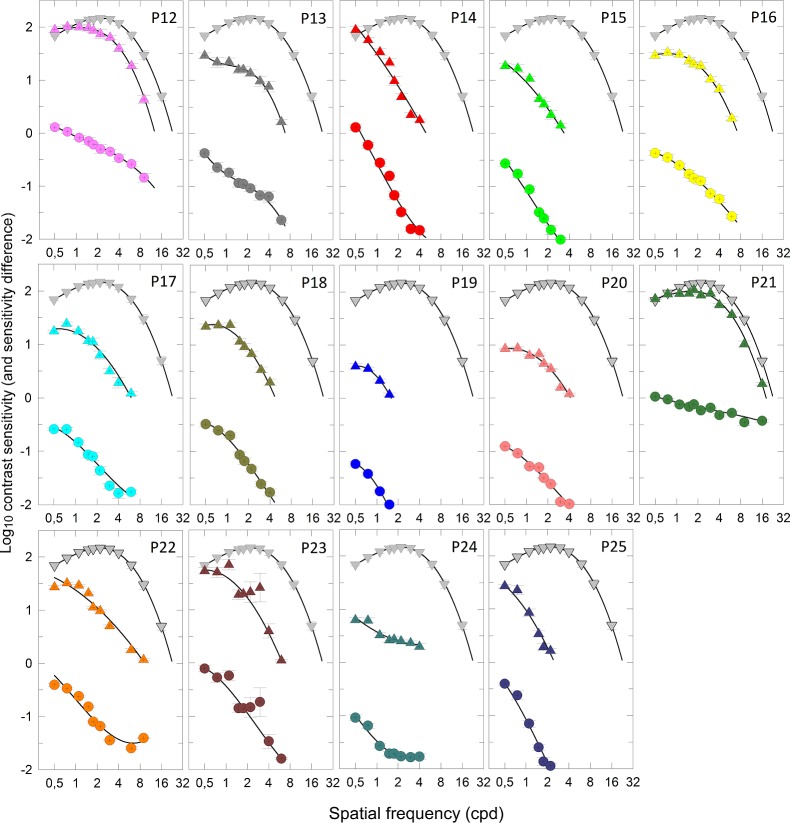
Continued.

**Figure 5:**
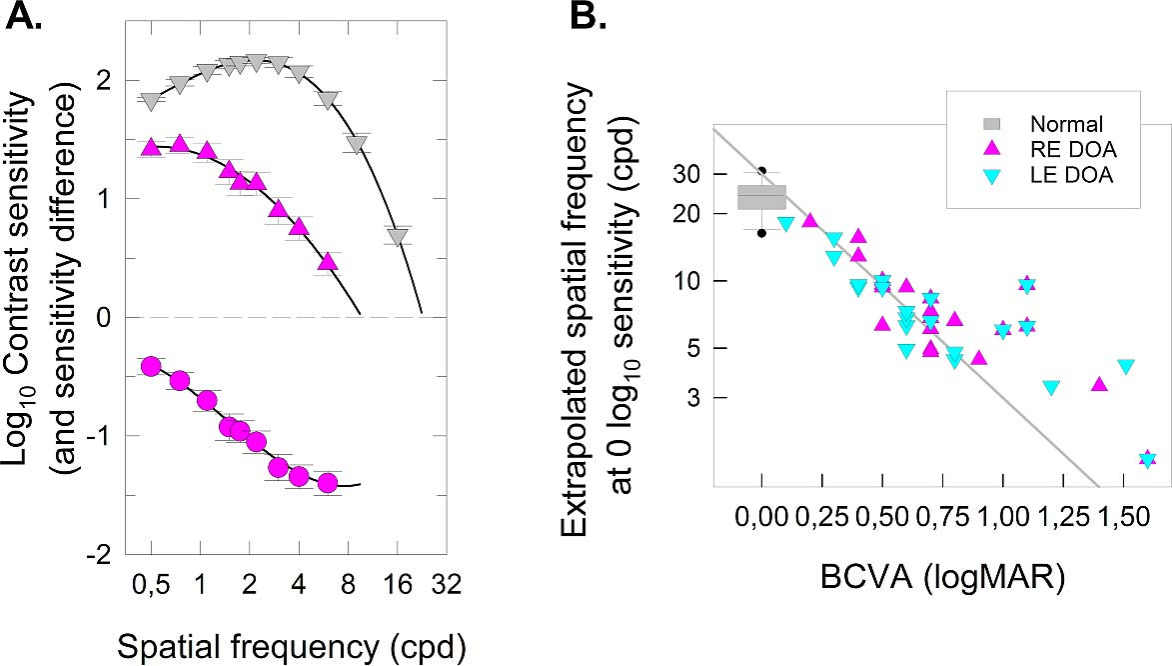
(**A**) Mean achromatic spatial contrast sensitivity function (CSF) of 25 DOA (*pink triangles*) and 15 normal observers (*gray triangles*) expressed in log_10_ sensitivity as a function of spatial frequency (cycles per degree, cyc/deg—logarithmic axis). (Extrapolation of the fitted SCSFs to zero sensitivity; that is, sensitivity corresponding to the maximum [100%] sinusoidal contrast, is determined by the spatial frequency at which the SCSFs intersect the *horizontal gray dashed line*.) The mean sensitivity difference between DOA patients and the mean normal observer is shown as *colored circles* in the *lower image*. The *symbols* and *error bars* are mean ± 1 SEM within both groups. The *black curves* are the model fits, as described in the text. (**B**) Spatial frequency (cyc/deg) at zero sensitivity (logarithmic axis) is plotted as a function of the best-corrected visual acuities (BCVA) of both eyes. The *gray line* denotes the correlation between the spatial frequency and the visual acuity expressed in logMAR units. The data for DOA patients are presented as a scatter plot, and data for normal observers as a box plot showing median, range (*error bars*), interquartile range (*box*), and an outlier (*black dots*).

The data for DOA P1 to P25 are shown with individually colored symbols as mean ± 1 SEM in the upper part of each image (colored triangles), together with normal data (inverted gray triangles). Differences in sensitivity between individual DOA patients and normal are shown as colored circles in the lower part of each image. There is little loss in sensitivity at low frequencies for the DOA patients, with the exception of P8, P9, P18, P19, and P24, but all DOA patients show substantial losses relative to normal observers at high spatial frequencies.

The fits of the cubic polynomial functions are shown as black curves in Figures 4 and 5. The maximum visible frequency is taken as the zero crossing of the cubic function, and the maximum sensitivity and peak frequency can be found as the local maximum in the cubic function, if it exists. Table 6 tabulates these values. Note that for nine patients (denoted by asterisk) the TSCF appears low-pass with no discernible peak in sensitivity, and in these cases, for analysis purposes, the peak frequency was defined as the lowest frequency tested (0.5 cyc/deg) and the peak sensitivity was simply the sensitivity at that frequency.

**Table 6:**
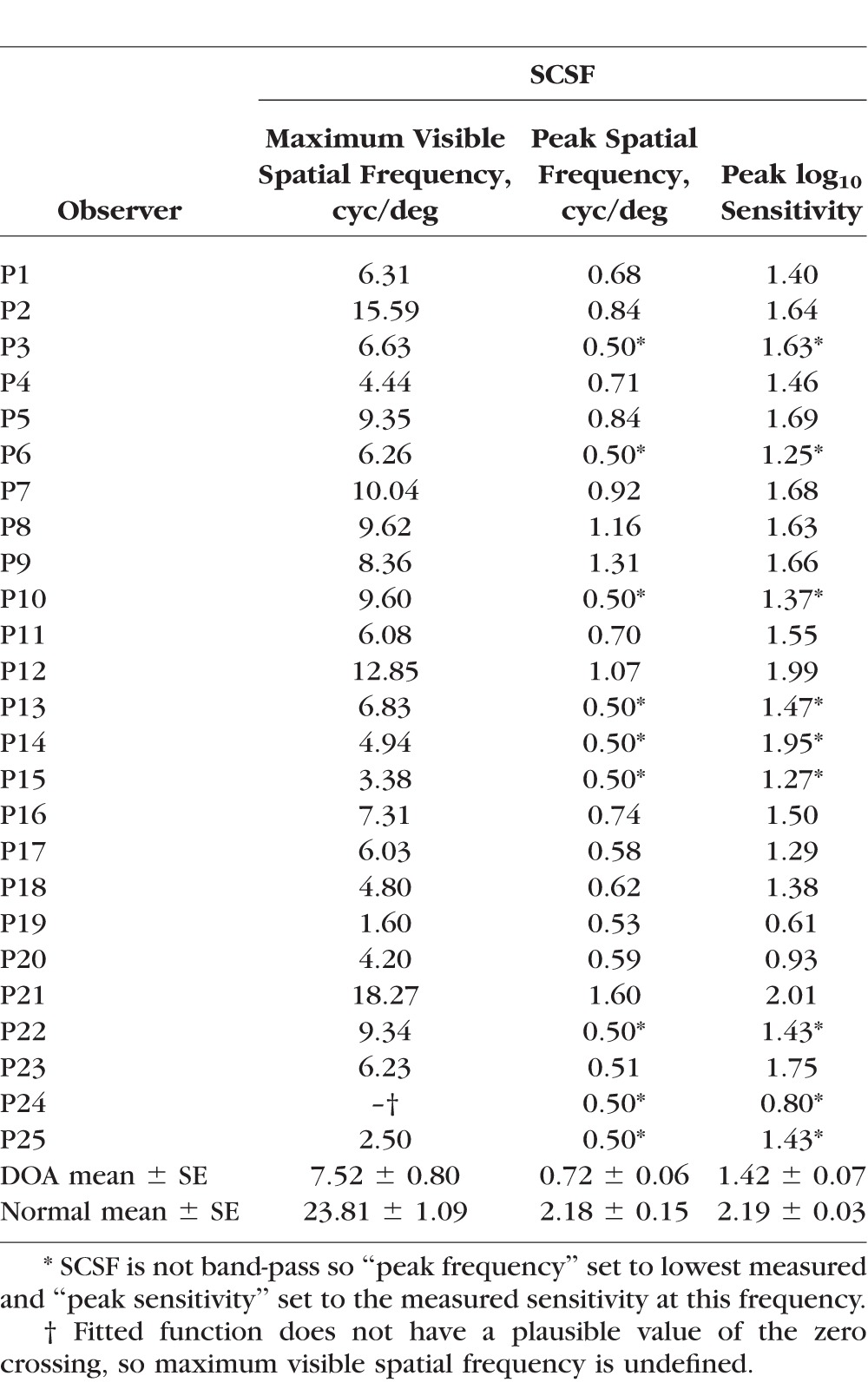
Peak and Maximal Sensitivity and Highest Visible SCSF Frequency

Figure 5A presents the mean achromatic SCSF for the entire 25 DOA patient group. The patients have a significantly lower achromatic spatial contrast sensitivity than normal with a mean peak sensitivity of 1.42 ± 0.07 log_10_ (mean ± SEM) shifted to the spatial frequency of 0.72 ± 0.06 cyc/deg (mean ± SEM). The sensitivity loss increased nearly linearly toward high spatial frequencies, and mean CSF function was low-pass or band-pass for the DOA patients instead of being consistently band-pass in the normal observers. The difference in peak sensitivity for patients compared to normal observers was not correlated with the sensitivity losses found in the TCSF data (Pearson's *r* = 0.284, *P* = 0.426).

The spatial acuity at maximum contrast derived from the model fits for each is plotted in Figure 5B. The highest observed spatial frequency under maximal contrast (corresponding to zero sensitivity) relates to spatial visual acuity expressed as logarithm of the minimum angle of resolution (logMAR), so that 0 logMAR corresponds to 30 cyc/deg.^49^ This value for spatial frequency corresponds to the extrapolated intercept of the contrast sensitivity function at zero sensitivity. Figure 5A shows the mean achromatic SCSF for DOA and normal observers and extrapolation of SCSF to zero sensitivity at the intersection with the horizontal gray dashed line. Figure 5B shows the spatial frequencies obtained by extrapolation of SCSF of each observer at zero sensitivity plotted as a function of BCVA expressed in logMAR units. In normal observers with normal visual acuities (0 logMAR), the extrapolated SCSF at zero sensitivity had median intercept at 23.8 ± 1.09 cyc/deg (median ± SEM). In the DOA patients with BCVA better than 1.0 logMAR, the SCSF intercepts correspond roughly with the BCVA values. The logMAR is a reasonable predictor of spatial acuity below 0.75, but not above it. There was no sex-related variation in the BCVA (Mann-Whitney *U* test, *P* = 0.210).

The achromatic SCSF has a spatial frequency–dependent contribution from several RGC subpopulations: (1) Midget cells with the smallest receptive field probably mediate the highest spatial frequencies^44,48^; (2) OFF midget cells may have more significant contribution at low spatial frequencies than ON midget cells^45^; (3) parasol RGCs are also sensitive to low spatial frequencies.^50^ We analyzed the statistical dependence between the nonspatial variables (age, the structural findings, visual field defect, temporal data, chromatic data) and the spatial losses at low (0.75 cyc/deg) and higher (4 cyc/deg) spatial frequencies separately. Losses at both frequencies, however, showed similar associations with the field and color vision defects and the structural variables, and no correlation with the temporal data (studied within the 11-patient subgroup) or dependence on age.

### Chromatic Discrimination

**Figure 6:**
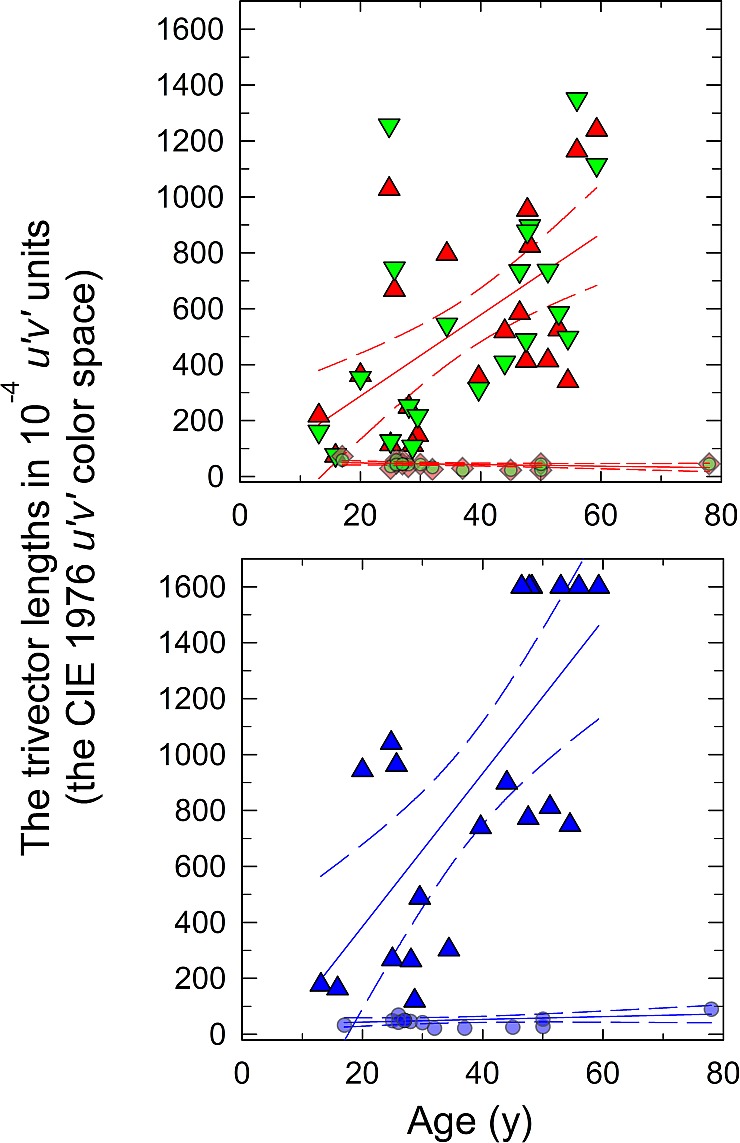
The Trivector Cambridge Colour Test. The vector lengths in the protan and deutan (**A**) and tritan (**B**) confusion lines expressed in 10^−4^ u′v′ units (the CIE 1976 u′v′ color space) as a function of observer age for 23 DOA (*colored symbols*: *upper image*, *red triangles* for protan, *inverted green triangles* for deutan, and *lower image*, *blue triangles* for tritan). Normal observers in both images (*small gray diamonds*). *Solid lines* are for the best linear regression and *dashed lines* for the 95% confidence intervals.

Figure 6 shows the vector lengths of the three confusion lines (protan, deutan, and tritan) of the CCT in 10^−4^ u′v′ units (the CIE 1976 u′v′ color space) for 21 DOA and 15 normal observers presented as a function of observer's age. The longer the vector length, the more saturated color was required for color discrimination, with the maximum saturation corresponding to the vector length of 1600 10^−4^ u′v′ units (the CIE 1976 u′v′ color space). In the normal observers (smaller partially transparent symbols), the vector lengths were similar to those previously reported using the standard long viewing distance with the gap in the letter C opening subtending 1° of visual angle: 45.1 ± 1.0 for protan, 43.3 ± 0.8 for deutan, and 51.5 ± 1.3 for tritan in 10^−4^ u′v′ units (the CIE 1976 u′v′ color space) (mean ± SEM).^51^ No significant age-related variation in any of the confusion lines was observed in normal observers.

Dominant optic atrophy patients had abnormal chromatic contrast sensitivity on all three confusion lines with the following mean vector lengths: 659 ± 94 for protan, 696 ± 94 for deutan, and 988 ± 111 for tritan in 10^−4^ u′v′ units (the CIE 1976 u′v′ color space) (mean ± SEM). Three DOA patients could not detect even the most saturated color in any of the confusion lines, and their data are not included in Figure 6. Two of them, aged 34 and 65 years, carried *OPA1* c.733A>T mutation, and the third patient, aged 39 years old, *OPA1* c.112C>A mutation. In addition, *OPA1* c. 1296delCAT mutation associated with early loss of red–green color vision, unrelated to the visual acuity or field losses, and with an equal loss of color discrimination in all three confusion lines, whereas in the other genotypes the tritan axis was always more severely affected. There was thus evident genotype-related variation in the chromatic discrimination. However, in general, color vision deteriorated with increasing patient age (Pearson's *r* = 0.545, *P* = 0.011 for protan, *r* = 0.581, *P* = 0.006 for deutan, and *r* = 0.710, *P* < 0.001 for tritan axes). There was no sex-related variation in the chromatic discrimination (Mann-Whitney *U* test, *P* = 0.891 for protan, *P* = 0.891 for deutan, and *P* = 0.945 for tritan axes).

For comparison of regression rates between color vision along the tritan confusion line and S-cone temporal acuity, data for the 11 DOA patients undergoing both tests were converted to percentage of the normal values and referred to the observer age. Both short wavelength–related visual functions showed similar regression (Pearson *r* = −0.734, *P* = 0.010 for tritan vector length, and *r* = −0.856, *P* = 0.001 for S-cone temporal acuity) corresponding to annual deterioration of 1.9% for both functions.

### OCT Structural Data

**Figure 7:**
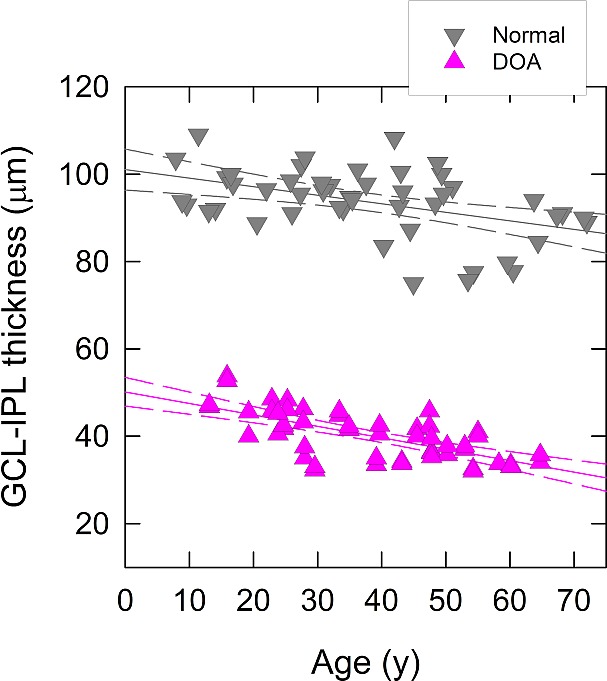
Macular ganglion cell–inner plexiform layer (GCL-IPL) thickness (μm) for 50 eyes of 25 DOA patients (*pink symbols*) and for 48 healthy eyes (*gray symbols*) plotted as a function of subject age and including lines for the best fits of linear regression and 95% confidence intervals.

The mean macular GCL-IPL thickness in 50 eyes of 25 DOA patients was 39.7 ± 1.10 μm (mean ± SEM), that is, 42% of the normal, 93.5 ± 1.4 μm (mean ± SEM). The mean peripapillary RNFL thickness was 61.9 ± 1.2 μm (mean ± SEM), corresponding to 63% of the normal and to that previously reported in another cohort of nonsyndromic DOA.^52^ Figure 7 shows the thickness of the GCL-IPL complex measured from the macular SD-OCT images of 50 eyes of 25 DOA patients (pink triangles) and of 48 normal eyes (gray inverted triangles) plotted as a function of age. Dominant optic atrophy patients had significantly thinner GCL-IPL complex than normal at all ages, but the best fit for linear regression of the GCL-IPL thickness both for normal subjects (Pearson's *r* = −0.470, *P* < 0.001) and for DOA patients (*r* = −0.685, *P* < 0.001) corresponded to 0.2 μm loss and 0.2% per year. Dominant optic atrophy patients did not show any sex-related variation in the GCL-IPL thickness (Mann-Whitney *U* test, *P* = 0.240).

## Discussion

Visual impairment in DOA was characterized by losses that can be associated with all three visual pathways, but with inhomogeneous severity and age-related variation. The loss of midget RGCs could account for the loss of high spatial frequency sensitivity that was markedly subnormal in most DOA patients at all ages. It could also account for the loss of chromatic resolution along the protan and deutan color confusion lines, which showed clear deterioration with increasing patient age. By contrast, the loss of parasol RGCs, which could account for the loss of high temporal frequency acuity, was mild and without significant age-related variation. The most striking age-related visual losses, however, were observed for the S-cone–mediated chromatic and temporal sensitivities, which could result from the loss of small bistratified RGCs.

Visual perception results from the sequential and parallel transmission and processing of signals from the photoreceptors through the visual system. In DOA, there is no evidence of functional or structural abnormalities in the outer retina^7,53–55^; and although a subgroup of patients carrying pathogenic *OPA1* mutations can develop a more severe neurologic phenotype and nonspecific white matter cortical abnormalities, the neuropathology in the majority of patients is limited to the optic nerve.^56^ The psychophysical abnormalities identified in our patient cohort could therefore be attributed to RGC loss or dysfunction. At this level of the visual pathway, perception depends on the parallel transmission of signals by different subpopulations of RGC types that encode different characteristics of visual target.^44,57^ In DOA patients, the parasol RGC–related visual functions showed a lowered temporal acuity, a slower increase in acuity with increasing radiance than normal, and a roughly constant sensitivity loss across temporal frequencies. Could these findings be attributed to loss of a particular population of RGCs (e.g., the parasol cells) or to functional abnormalities within surviving members of that population? Psychophysical studies following a magnocellular lateral geniculate lesion in macaques revealed TCSF losses that increase with temporal frequency, but with some preservation of low frequency sensitivity, thus changing the shape of the TCSF from band-pass to low-pass.^58,59^ The pattern of magnocellular visual function in DOA differs from that reported for the anatomic loss of the magnocellular pathway. In contrast, nearly identical temporal losses to that observed in DOA have been described for single parasol RGC firing pattern in experimental primate glaucoma.^60^ In this model there was loss of the temporal acuity by 10 Hz, attenuation of the temporal sensitivity in the most dysfunctional RGCs also at low frequencies, and preservation of the band-pass sensitivity function. The extent of the functional abnormalities correlated with the loss of normal RGC dendritic arborization that is thought to represent the first sign of RGC degeneration in glaucoma.^61^ A similar structural feature has also been found in the B6;C3-*Opa1*^Q285STOP^ mouse model as a precursor of RGC degeneration.^62^ The architecture of dendritic arborization in patients with *OPA1* mutations is not known, but our data support the idea that loss of dendritic arborization may be involved in the pathogenesis of DOA rather than just population loss.

Previous psychophysical studies in nonhuman primates on the effect of the parvocellular lateral geniculate lesion have shown losses of the high and low spatial frequency contrast sensitivity and a change from a band-pass to a low-pass SCSF consistent with detection by the magnocellular pathway.^63^ Achromatic spatial contrast sensitivity in our DOA patient cohort had similar characteristics. However, experimental models of parvocellular pathway lesion also resulted in complete loss of color vision,^58^ which was observed in only a few DOA patients carrying a particular genotype (genotype 10, c.733A>T), or with the most advanced disease (genotype 11, c.112C>A). This suggests that the parvocellular lesion in DOA preserves sufficient midget RGCs to support chromatic resolution along the protan and deutan axes,^64^ but because of progressive loss there is also a steep age-related regression. Our results are in line with the report by Reis and colleagues^28^ that showed increasing contrast thresholds at higher spatial frequencies for their DOA patients, although less than half of their cohort carried confirmed pathogenic *OPA1* mutations. Our study also supports the structural and functional evidence pointing toward the midget cells as being the mainly affected RGC type in DOA, such as the predominance of the midget cells in the macular RGC population subserving central vision, and the preferential loss of the small-diameter axons in postmortem histopathologic studies^65,66^ and in a recent *OPA1* mouse model.^67^ Toxic and inflammatory insults to midget RGCs do not result in loss of mainly the high spatial frequencies,^68–70^ which implies that the preferential loss of the small receptive field midget cells may be specific to DOA.

The most extensive visual impairment was loss of S-cone chromatic and temporal sensitivities in the oldest DOA patients. This observation could be related to dysfunction of the small bistratified RGCs, which support S-cone vision (see above). Unlike normal age-matched observers, this group of DOA patients had unmeasurable chromatic resolution along the tritan confusion line and they were unable to detect flickering short-wavelength light. Loss of perception of the blue colors in DOA was also among the early observations by Kjer^7^ and the most frequently reported color vision defect in this disorder.^1,4,25–29^ Reports showing clear age-related variations are, however, scarce; and this could be explained by the differences in the testing conditions used. The Trivector test procedure of the CCT enables better discrimination and quantitative assessment of the individual color axes than the elliptical test procedure previously used in another study.^27^ Furthermore, our modifications of the viewing distance and response time, in turn, enabled use of the test for patients with varying impairment of the spatial resolution. Temporal sensitivity test of the koniocellular pathway (i.e., S-cone CFF) introduced an independent method for the assessment of the small bistratified RGC function. Importantly, this modality showed loss with increasing patient age identical to that observed for chromatic sensitivity.

A cross-sectional study does not allow disease progression to be followed at the individual level, but a prospective long-term longitudinal study in DOA is challenging. Despite these caveats, we carefully designed our study to include DOA patients covering a broad range of disease duration, and this approach enables us to speculate on a particular pattern and chronology of RGC loss in this disorder. Firstly, the macular GCL-IPL thickness in DOA patients was less than 50% of normative values and with a similar rate of decline with increasing age compared with healthy controls. Similar findings have been reported in other DOA cohorts,^9–11^ and taken together, the evidence indicates that a major proportion of the RGC loss has already occurred in early childhood, and probably starting in utero. If the remaining 50% of the GCL-IPL thickness is liberally converted to RGC density, that is, 50% of the foveolar maximum, this corresponds approximately to the RGC density found at a foveolar eccentricity of 7° in normal retina.^48^ Such an RGC density enables spatial resolution of 9 cyc/deg,^48^ which could be used as an estimate for the theoretical upper limit of spatial visual acuity in DOA. Interestingly, in the majority of our DOA patients, the spatial acuities were of that level, or below, leading to an interpretation that the high-density small receptive field midget RGCs could constitute the majority of the 50% RGC population that has already been lost.

Parasol and small bistratified cells form only a small proportion of the total RGC population, and detecting their loss may not be possible with current in vivo imaging techniques, requiring instead more sensitive functional psychophysical testing methods. Our results indicate that parasol RGCs are functionally compromised in DOA, but there is no clear evidence of continuing cell loss. In contrast, the functioning of the koniocellular S-cone pathway seems to rapidly deteriorate with increasing patient age, an observation that is consistent with the ongoing loss of small bistratified RGCs. The majority of patients with DOA will experience gradual loss of visual function, and although this provides an extended window of opportunity for therapeutic intervention, designing cost-effective clinical trials to detect a favorable outcome measure within a reasonable time frame is challenging. Although a prospective longitudinal study is needed to confirm our findings for individual *OPA1* mutation carriers, monitoring koniocellular pathway function with S-cone–mediated chromatic and temporal sensitivities could potentially provide useful biomarkers of disease progression in DOA.
